# tRNS boosts visual perceptual learning in participants with bilateral macular degeneration

**DOI:** 10.3389/fnagi.2024.1326435

**Published:** 2024-02-21

**Authors:** Giulio Contemori, Marcello Maniglia, Jade Guénot, Vincent Soler, Marta Cherubini, Benoit R. Cottereau, Yves Trotter

**Affiliations:** ^1^Department of General Psychology, University of Padova, Padua, Italy; ^2^Centre de Recherche Cerveau et Cognition, Université de Toulouse, Toulouse, France; ^3^Department of Psychology, University of California, Riverside, Riverside, CA, United States; ^4^Centre National de la Recherche Scientifique, Toulouse, France; ^5^Service d’Ophtalmologie Centre Hospitalier Universitaire de Toulouse, Toulouse, France; ^6^Department of Psychology and Cognitive Science, University of Trento, Rovereto, Italy

**Keywords:** macular degeneration, perceptual learning, tRNS, contrast detection, transfer of learning

## Abstract

Perceptual learning (PL) has shown promise in enhancing residual visual functions in patients with age-related macular degeneration (MD), however it requires prolonged training and evidence of generalization to untrained visual functions is limited. Recent studies suggest that combining transcranial random noise stimulation (tRNS) with perceptual learning produces faster and larger visual improvements in participants with normal vision. Thus, this approach might hold the key to improve PL effects in MD. To test this, we trained two groups of MD participants on a contrast detection task with (*n* = 5) or without (*n* = 7) concomitant occipital tRNS. The training consisted of a lateral masking paradigm in which the participant had to detect a central low contrast Gabor target. Transfer tasks, including contrast sensitivity, near and far visual acuity, and visual crowding, were measured at pre-, mid and post-tests. Combining tRNS and perceptual learning led to greater improvements in the trained task, evidenced by a larger increment in contrast sensitivity and reduced inhibition at the shortest target to flankers’ distance. The overall amount of transfer was similar between the two groups. These results suggest that coupling tRNS and perceptual learning has promising potential applications as a clinical rehabilitation strategy to improve vision in MD patients.

## 1 Introduction

Macular Degeneration (MD) is an ocular disease that affects the central part of the retina and causes central vision loss. It currently represents the main cause of visual impairment in the western world (Wong et al., 2014). Late-stage MD patients tend to show a preference for a specific peripheral portion of their spared retina, the preferred retinal locus (PRL), as a replacement for their fovea (Crossland et al., 2005; Riss-Jayle et al., 2008; Gheorghe et al., 2015). However, peripheral vision differs in many ways from central vision, with unstable fixation and poorer processing of finer visual details (Hogg and Chakravarthy, 2006; Macedo et al., 2011). In particular, the functional organization of cortical regions coding for peripheral vision is very different from that of regions connected to the fovea, with larger neurons’ receptive fields (Virsu and Rovamo, 1979), different distribution of photoreceptors (Elsner et al., 2017) and the peak of contrast sensitivity shifted from high spatial frequencies in the fovea to low spatial frequencies in the periphery (Virsu et al., 1982; Wright and Johnston, 1983). Because of these differences, basic everyday activities are very hard to perform for individuals with central vision loss (Battista et al., 2005). As MD is projected to affect over 288 million people worldwide by 2040 (Wong et al., 2014), developing rehabilitation strategies is of crucial importance for public health. Although researches in genetic therapies and retinal implants made incredible progress over the last years, there are currently no therapies to restore a damaged fovea (Gehrs et al., 2006; Makin, 2019). Treatment options focus on the use of visual aids coupled with various types of training which allow patients to partially compensate for their central vision loss (Maniglia et al., 2016). Recently, attention has been given to the use of Perceptual Learning to improve residual vision in the PRL (Chung, 2011; Plank et al., 2014; Maniglia et al., 2016, 2018, 2020). Perceptual learning (PL), a training regime based on the repetition of simple visual tasks such as contrast detection or orientation discrimination, has been successful in improving visual abilities in pathologies caused by refractive problems [myopia (Camilleri et al., 2014), presbyopia (Polat et al., 2012; Lev et al., 2014)] and atypical development [amblyopia (Polat et al., 2004; Barollo et al., 2017)], thereby emerging as a promising therapeutic approach (Dosher and Lu, 2017). However, PL effects in MD patients have not been equally successful, particularly in terms of transfer of learning (Chung, 2011; Plank et al., 2014). Possible reasons for this are the reduced cortical plasticity associated with the elderly age of most MD patients (Freitas et al., 2011; Lambert et al., 2016), and the structural differences between fovea and periphery.

Maniglia et al. (2016, 2020) trained MD participants on contrast detection with lateral masking, a protocol which led to a transfer of learning in both healthy and clinical populations trained in the fovea (Polat, 1999; Polat, 2009) and in the near periphery (Maniglia et al., 2011). Lateral masking displays are usually composed of a triplet of vertically aligned Gabor patches, whose central element is a low contrast target while the two flankers are high contrast. Depending on the target-to-flanker separation, usually expressed as a multiple of the target wavelength (λs), this modulation can be inhibitory (decrease of contrast sensitivity for the central target) or facilitatory (increased sensitivity for the target). Prolonged training with both inhibitory and facilitatory separations improves contrast sensitivity for the central target over multiple target-to-flanker separations (Polat and Sagi, 1994). It has been suggested that the mechanisms underlying the transfer of learning observed in studies using lateral masking (Polat, 1999, 2009; Maniglia et al., 2011; Lev et al., 2015) lie in the neural substrates responsible for this effect, which is thought to be the horizontal connections between neurons sharing similar spatial frequency and orientation tuning in early visual cortex (Ts’O et al., 1986; Gilbert and Wiesel, 1989; Grinvald et al., 1994). Improving neural responses at the first stages of visual processing would then provide higher visual regions with better inputs, improving in turn higher visual functions such as visual acuity (Polat, 2009) and visual crowding (Maniglia et al., 2011).

Results from Maniglia et al. (2016) showed transfer of learning to visual acuity and contrast sensitivity at untrained spatial frequencies. However, unlike what was observed in healthy participants (Maniglia et al., 2011), MD participants did not reduce their visual crowding. Patients may need longer training to achieve the desired transfer, but on the other hand, excessive training may increase training specificity (Jeter et al., 2010). An alternative way to achieve the desired transfer could be to promote participants’ neural plasticity during training. Recent studies have shown that under some conditions, non-invasive brain stimulation [NIBS], an easily accessible neuro-modulatory technique, can increase the visual improvement derived from repetitive training (Koganemaru et al., 2015), promoting visual recovery in multiple diseases (Sabel et al., 2020). Transcranial random noise stimulation (tRNS), a type of randomly alternating current stimulation with frequencies spanning from 100 to 640 Hz, has been used to boost perception and learning in both healthy participants and clinical populations (Campana et al., 2014; Contemori et al., 2019; Herpich et al., 2019; Donkor et al., 2021). In a recent study, we tested the effects of tRNS coupled with peripheral perceptual training in normal sighted participants (Contemori et al., 2019). Participants were trained with a crowded letter recognition task with or without stimulation. Training effects were larger in the tRNS group than in the sham group, while both achieved similar levels of transfer. In another study conducted by Moret et al. (2018), tRNS increased transfer to visual acuity. Taken together, these results suggest that tRNS can boost training effects.

The present study aimed to test whether tRNS coupled with perceptual learning can increase learning and transfer in participants suffering from central vision loss. To this end, we trained two groups of MD participants on contrast detection with lateral masking, a low-level task that might facilitate learning transfer to higher visual functions (Maniglia et al., 2018; Moret et al., 2018). The two groups of MD participants underwent the training with and without online tRNS, respectively. Given that early visual areas seem to be the neural substrates of lateral masking (Polat and Norcia, 1996; Mizobe et al., 2001; Cass and Alais, 2006), we chose the occipital lobe as the locus of stimulation. Assessment tasks measuring a range of visual functions were conducted before, halfway through, and at the end of the training. This included contrast sensitivity, near and far visual acuity, and visual crowding.

## 2 Materials and methods

### 2.1 Apparatus

Stimuli were displayed on a 17″ Dell M770 CRT monitor with a resolution of 1,024 × 768 pixels, a refresh rate of 60 Hz, and a mean luminance of 47.6 cd/m^2^. Except for the visual acuity task, stimuli were generated with Matlab Psychtoolbox (Brainard, 1997). Each pixel subtended 2.14 arcmin of visual angle. For tasks involving contrast sensitivity measures, a digital-to-analog converter (Bits#, Cambridge Research Systems, Cambridge, UK) was used to increase the dynamic contrast range (13-bit luminance resolution). The monitor was linearized thanks to a 12-bit gamma-corrected lookup table (LUT). Participants sat in a dark room at 57 cm from the screen (200 cm for the visual acuity test). A chin rest was used to keep the participants’ heads at the right distance. Experiments were carried on at the Centre de la Retine, Hôpital Pierre-Paul Riquet, Purpan Hospital, Toulouse (France).

### 2.2 Participants

12 MD participants took part in the study. The data and training results from 5 of these participants (all in the ‘PL – only’ group) were also included in a previous study (Maniglia et al., 2018). Candidate participants were selected from the list of patients in the ophthalmology service of the Toulouse Retina Center (Pierre Paul Riquet Hospital). The first contact was made through their ophthalmologist. If they expressed interest in the study, they were invited to the hospital for a free ophthalmological assessment on the basis of which their inclusion was evaluated. All participants gave their written informed consent prior to their inclusion. This study followed the ethical standards of the Declaration of Helsinki (World Medical Association, 1996), and the experimental protocol was approved by the CNRS ethical committee (Comité de Protection des Personnes, protocole 13,018). Participants were reimbursed for all the travel expenses related to their participation.

Only participants with an absolute central bilateral scotoma and a residual visual acuity between 1/10 and 3/10 were included in the study. This inclusion criterion had a dual purpose. On the one hand, it served to ensure that the participants had sufficient visual resolution to see the stimulus triplet clearly in the easiest condition (high contrast, 1 cpd). On the other hand, it allowed excluding participants with residual central retinal islands with high visual acuity. A visual field test was performed in both eyes by means of an Octopus^®^ 300 perimeter, Köniz, Switzerland. The training was performed monocularly in the eye with best visual acuity and fixation stability. In patients with bilateral scotoma, there is no complete overlap between the two retinal lesions, and one eye may have much better visual residual than the other. Monocular training ensured that the training stimulus fell entirely outside the scotoma, also avoiding the possible defocus introduced by the worse eye. Moreover, we only included participants with a single and (fairly) stable PRL in the trained eye. It should be noted that some patients show different PRLs – in the same eye – in binocular versus monocular vision (Déruaz et al., 2002; Tarita-Nistor et al., 2015). Monocular testing also allowed a better match with the PRL localization obtained by Optical Coherence Tomography (OCT). Stability of PRL was assessed by acquiring at least three different images during the OCT with pauses in between each acquisition. For each image the PRL was localized, and the resulting positions compared with each other. The PRL was considered as stable if the variance between different acquisitions was no more than 3 deg. The presence of concomitant ocular diseases or a non-stabilized PRL was considered a ground for exclusion. We also excluded participants with diagnosed cognitive or mood disorders. Based on these criteria, only 16 were eligible after the initial ophthalmological screening. 12 completed the study. Of the remaining 4, one retired after a week of training for personal reasons. One underwent chemotherapy shortly after joining the study and was therefore excluded. Two were excluded right after the pre-tests as they reported that they could not see all of the three stimuli in the training configuration. Data were collected over 5 years, first in the PL-only group and subsequently in the PL + tRNS group.

### 2.3 PRL localization

**Figure 1:**
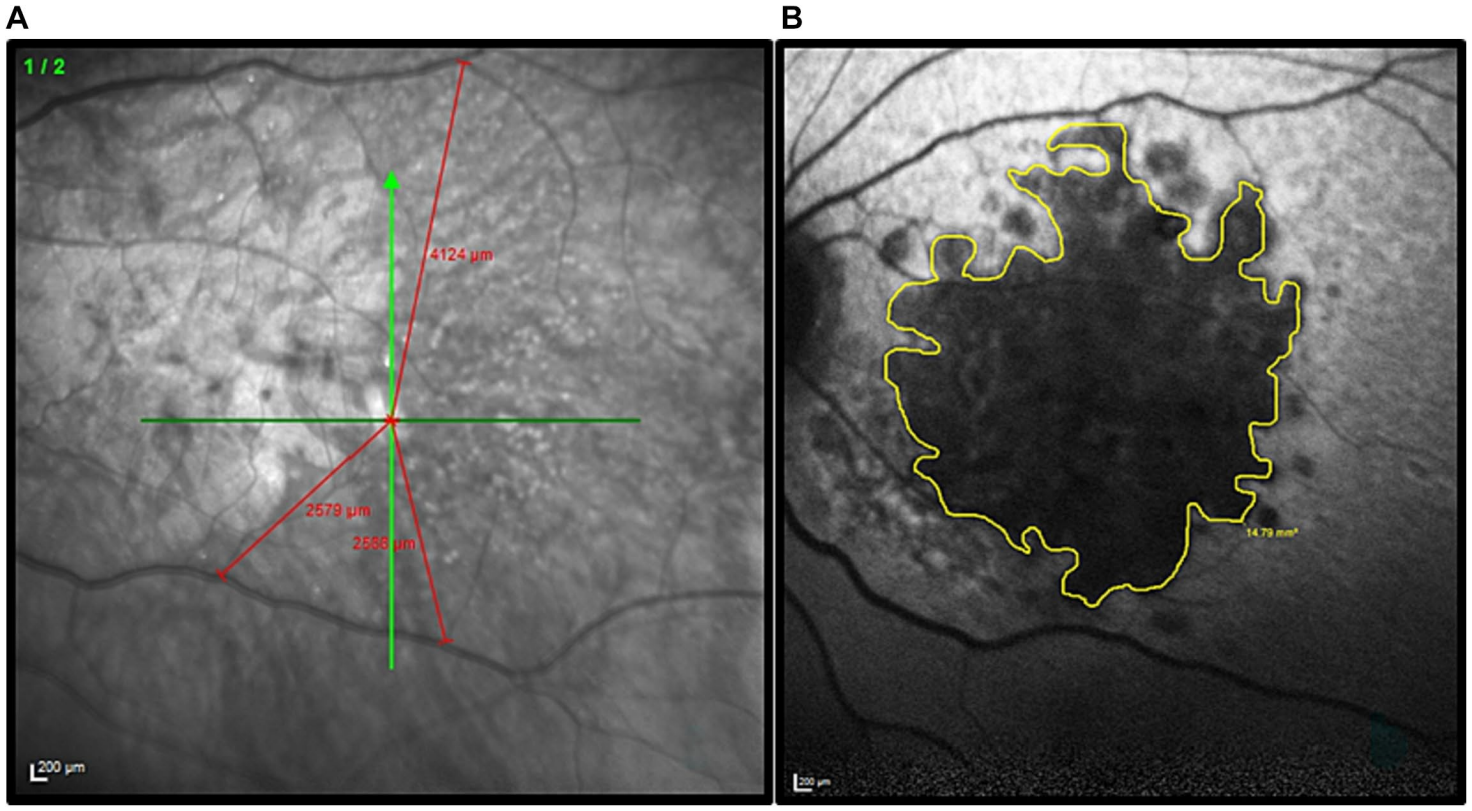
Illustration of the procedure used to define the PRL position and delineate the border of the retinal lesion in each MD patient. **(A)** Example of the trilateration of the PRL through the three anatomical landmarks. The green cross indicates the PRL position. The distance PRL/landmarks are highlighted in red. **(B)** The patient’s retinal lesion is highlighted in yellow before the diameter calculation.

For each participant, the position of the PRL was determined following the trilateration procedure described by Maniglia et al. (2018) (see also Guénot et al., 2022). First, the fovea was localized through a high-resolution scan of the retinal fundus with a Spectral-Domain Optical Coherence Tomography (Spectralis OCT, Heidelberg Engineering, Heidelberg, Germany). Three anatomical landmarks were then selected on the retinal fundus image and their coordinates were calculated with respect to the fovea. Presumed position of the fovea was inferred using the remnants of the foveal depression and foveal bulge in the cross-sectional OCT image. When it could not be located by image inspection, the presumed position of the fovea was estimated based on the average position relative to the optic disc. On average, the human fovea is located 6.3° ± 3.0° vertically below the optic disc (Rohrschneider, 2004). Later, the three landmarks were used to trilaterate the position of the retinal locus that corresponded to the fixation cross during the OCT acquisition. The three landmarks were individually chosen according to the participant’s retinal blood vessel topography (blood vessels bifurcations). They had to be clearly visible in all retinal scans. To check for the presence of multiple PRLs, the procedure was repeated three times. PRL coordinates were calculated with reference to the assumed position of the former fovea. The edge of the retinal lesion was automatically extracted based on the contrast difference between the lesion and healthy retina on the OCT image. Then through a manual procedure the perimeter was re-adjusted to better follow the irregular lesion borders. As an example, an image collected during this procedure is shown in Figure 1. Only participants who had a consistent PRL position across the three independent measurements were included in the study. The procedure was repeated during both the mid-test and the post-test to ensure that the PRL position was stable all over the training. Details of the participants are reported in Table 1.

**Table 1:** Details of the MD patients who participated in the study.

Participant	Gender	Age	Lesion diameter	Position of PRL x° y°	Tested eye	VA
MD1	Male	61	17°	Left-up-8.0° 0.2°	LE	1/10
MD2	Female	59	17°	Left-down-5.5° 1.0°	LE	1/10
MD3	Female	82	22°	Left-down-2.6° 9.1°	RE	2/10
MD4	Female	53	10°	Left-down-8° 1.5	LE	2/10
MD5	Female	77	12°	Left-down-9° 5°	RE	1/10
MD6	Female	89	16°	Left-up-6.5° -6.5°	LE	1/10
MD7	Female	81	20°	Left-down-3° 6.4°	LE	2/10
MD8	Female	70	17°	Left-down-9.8° 5.5°	LE	1/10
MD9	Female	67	14°	Left-Down-0.1° 4.7°	LE	2/10
MD10	Male	71	16°	Left-down-10.3° 3.8°	LE	1/10
MD11	Female	89	15°	Right-down-7.8° 1°	LE	2/10
MD12	Female	79	15.3°	Down-0.1° 6.9°	RE	1/10

Patients from 1 to 5 (in white) were trained with PL – only and were also included in Maniglia et al. (2018). Patients from 6 to 12 (in gray) were trained with concomitant tRNS stimulation. Retinal lesion diameter along the largest diagonal. PRL coordinates are reported using the assumed position of the former fovea as the center.

### 2.4 Procedure

**Figure 2:**
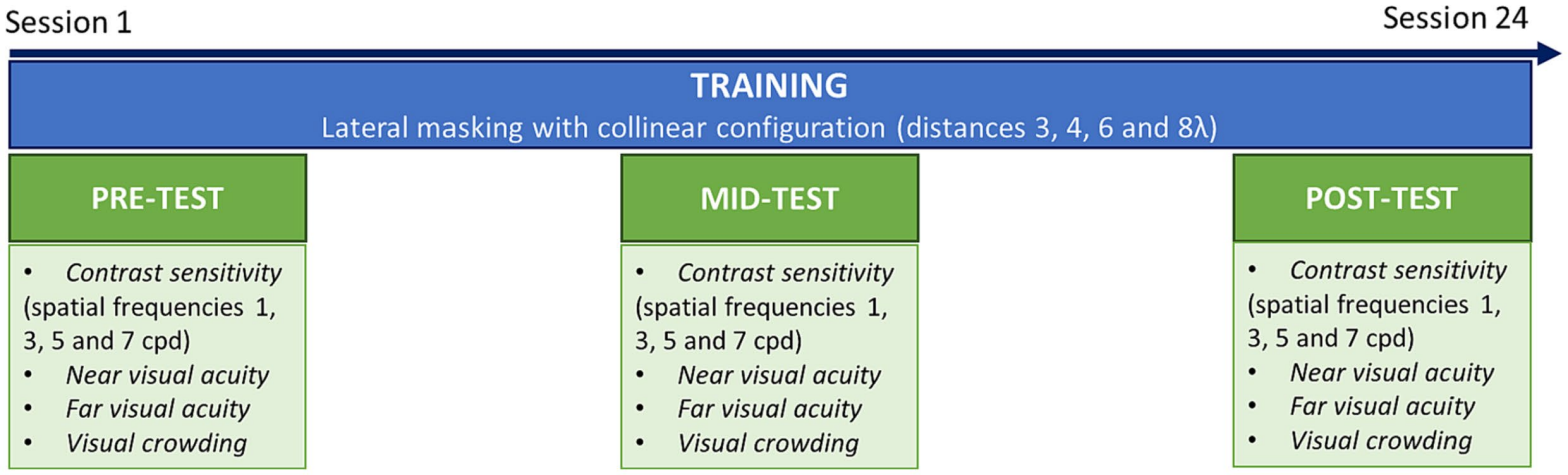
Experimental procedure. Patients were trained on a lateral masking task with collinear configuration, three times a week during 8 weeks (24 sessions in total). Before training (pre-test), after the 12th session (mid-test) and after the last session (post-test) transfer tasks were conducted, including contrast sensitivity, near and far visual acuity and visual crowing.

During pre-tests, mid-tests and post-test, participants underwent a series of assessment tasks, including contrast sensitivity, near and far visual acuity, and visual crowding (Figure 2). The training consisted of a lateral masking task with a collinear configuration. Each training session included 4 blocks and lasted ~25 min. Following previous studies with a similar configuration (Polat and Sagi, 1994; Polat, 2009; Maniglia et al., 2011), we trained participants over several target-to-flanker distances, which were manipulated between blocks. We used distances of 3, 4, 6, and 8λ (multiples of the stimulus wavelength). In the tRNS group, a concomitant stimulation was applied over the occipital cortex during the whole training sessions. Each participant completed three sessions a week during 8 weeks for a total of 24 training sessions. Mid-tests were conducted after 12 sessions. Post-tests were collected at the end of the training (after 24 sessions).

#### 2.4.1 Contrast sensitivity

Contrast sensitivity (CS) was measured for 4 different spatial frequencies using a custom-made MATLAB script. The target stimulus consisted of a single Gabor patch of 4 deg (full width at half maximum) with a vertical orientation. The procedure was a temporal-2AFC (two-alternative forced-choice) in which the target Gabor patch was only displayed during one of two intervals. Participants had to report the interval containing the target by pressing “1” or “2” on the keyboard. Each interval lasted 133 ms and the interstimulus separation was 500 ms. Target contrast varied according to a 3down/1up staircase (Levitt, 1971), in which three consecutive correct responses reduced the target contrast of 0.1 log units and each incorrect response increased the contrast of the same amount. The staircase terminated after 120 trials or 14 reversals. The procedure returned the 79% contrast threshold estimated from the algebraic mean of the 6 last reversals. A 50 ms-long acoustic cue signaled the beginning of each interval. Each spatial frequency was tested twice, once for each eye monocularly. The tested spatial frequencies were 1, 3, 5, and 7 cpd. The starting contrast was set to 30% at 1 cpd and was adapted to the participant’s performance for all the other spatial frequencies. The spatial frequencies were tested in ascending order. In the case where thresholds exceeded 90% during a recording block, the testing was interrupted. The new trial started only after the participant’s response was recorded. To facilitate ocular fixation, a 0.5 deg. dot was present in the center of the screen during the whole procedure. Participants were instructed to re-center their fixation on the dot after each trial. The testing was conducted monocularly for both eyes.

#### 2.4.2 Far visual acuity

To test far visual acuity (FVA), we used the FrACT (Freiburg Visual Acuity and Contrast Test) software (Bach, 1996), a computerized letter recognition task. This software is very robust and was successfully used in over 100 studies. It was also used in different clinical populations (Wesemann, 2002; Bach, 2006). Stimuli were randomly selected among 10 Sloan letters: C, D, H, K, N, O, R, S, V, and Z. Participants reported the letter aloud and the experimenter, sitting at a position from where the screen was not visible, typed the appropriate letter on the keyboard. Visual acuity measurements were collected monocularly for each eye. Threshold was calculated by means of an adaptive staircase procedure implemented in the FrACT software, the Best-PEST algorithm. Participants viewed the letters for a maximum of 30s at a viewing distance of 200 cm over 30 trials. Letters were black on a white background. Acoustic feedback was provided for both correct and incorrect answers. The average duration of each block was around 5 min. The testing was conducted monocularly for both eyes.

#### 2.4.3 Near visual acuity

Near visual acuity (NVA) was measured with a custom-made MATLAB script on a standard desktop computer. The stimuli were presented on a CRT monitor. Participants were instructed to fixate with their preferred retinal locus. In case of a loss of fixation, they had to memorize the last answer, search for the fixation dot, and then provide the verbal answer to the experimenter only once ready for the next trial. At this point, the experimenter reported the answer by pressing the corresponding button on the keyboard and the next trial was initiated. Randomly selected Sloan letters (D, N, S, C, K, R, Z, H, O, V) were used as stimuli and presented in the center of the screen for 100 ms. Letters were white on a black background. The starting letter size was 3 deg, a size fairly above the threshold for all participants. In the successive trials, the size changed following a psychophysical adaptive procedure from the MLP toolbox (Grassi and Soranzo, 2009), estimating a 75% threshold (Green, 1990; Green, 1993; Grassi and Soranzo, 2009). The block was composed of 30 trials, an adequate number to obtain a reliable and fast threshold (Leek et al., 2000). Threshold estimation was based on a logistic function defined by three fixed and a free parameter. The function’s slope (beta), the chance level (gamma) and the lapse rate (lambda), were set, respectively, to 0.5, 0.1 and 0. The free parameter corresponded to the displacement of the midpoint of the function along the abscissa (alpha). The threshold was defined as the size of the letter that would result in 75% accuracy.

A practice dummy block was performed at the beginning to help the participant to familiarize with the very fast presentation speed. During this dummy block, the experimenter performed the task together with the participant. During the actual measurement, the experimenter sat at a side of the desk, in a position from where she/he could not see the monitor displaying the stimuli. Participants were instructed to respond even when they could not see the letter. A fixation dot was displayed in the center of the screen. The dot disappeared right before the target onset and then reappeared right after the target offset. An acoustic cue preceded the stimulus onset of 100 ms and lasted for 50 ms. The procedure was self-paced, in a sense that the next trial did not start before the answer to the previous one was collected. To avoid lapses, participants were told that no time limit was applied to the answer, and they were also instructed to think carefully before answering. The total duration of the procedure was considerably variable between participants, but it never exceeded 3 min. The testing was conducted monocularly for both eyes.

#### 2.4.4 Visual crowding

Visual crowding was estimated using the same procedure as for the near visual acuity, except that we measured critical space instead of letter size. A triplet of letters was presented and the distance between the central target and the flanking stimuli varied. The threshold was defined as the critical distance that allowed for an accuracy of 75%. For calculating crowding, a procedure analogous to NVA was employed, using the adaptive procedure of the MLP toolbox (Grassi and Soranzo, 2009). To disentangle crowding measurements from visual acuity, the size of the three letters was kept constant and corresponded to 130% of the letter-size threshold obtained in the near visual acuity task. The testing was done monocularly for both eyes. To make sure that the participants could see all the three letters, the global orientation of the triplets could be horizontal or vertical, depending on the size and the shape of the retinal lesion. Letters were always vertical. The testing was conducted monocularly in both eyes.

#### 2.4.5 Training procedure

**Figure 3:**
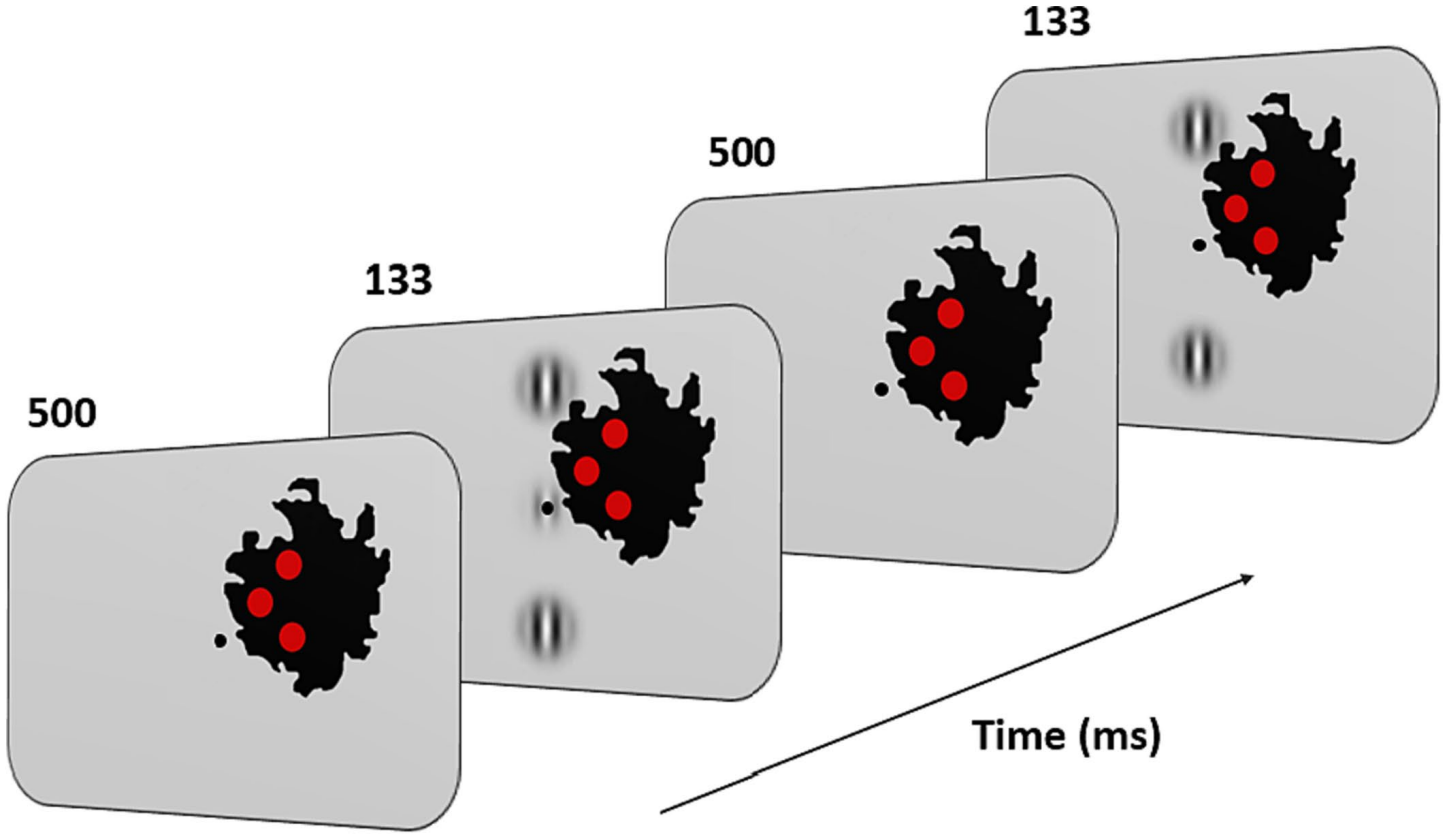
Experimental paradigm and training configuration. Patients had to fixate monocularly the point in the center of the screen with their PRL. Fixation stability was reinforced by three red disks displayed along the inner border of the scotoma (in black on the figure; note that this scotoma corresponds to that of the patient shown in Figure 1). Their positions were based on OCT measurements and on the subjective report of the patients. Under correct fixation on the center of the screen (the target location in the figure), the three disks were not perceived. When any of them became visible, patients were instructed to reallocate their gaze (i.e., their PRL) toward the center. During the task, two or three Gabors appeared at this central position for 133 ms with an inter stimulus interval of 500 ms. Patients had to report which interval contained the target (i.e., the low contrast central Gabor).

Training procedure was mutuated from Maniglia et al. (2018). Training stimuli were Gabor patches consisting of a cosinusoidal carrier enveloped by a stationary Gaussian. Each Gabor patch was characterized by its sinusoidal wavelength (λ), phase (*φ*), and standard deviation of the luminance Gaussian envelope (λ) in the (x,y) space of the image, where σ equaled λ and φ equaled 0 (even symmetric). Equation 1 illustrates the Gabor formula:


(1) $$G\left(x,y\right)=\cos\left(\frac{2\pi }{\lambda }x+\varphi \right)e^{\left(-\frac{x^{2}+y^{2}}{\sigma^{2}}\right)}$$

with λ = λ and *φ* = 0 (even symmetric). Gabors’ spatial frequency (SF) was 1 cpd, a frequency shown to maximize peripheral facilitation magnitude (Maniglia et al., 2015). A vertical low-contrast Gabor target (Figure 3) was collinearly flanked above, and below, by two iso-oriented high-contrast Gabors (0.7 Michelson contrast). Participants were trained at 4 different target-to-flanker separations: 3, 4, 6, and 8λ. Task consisted in a temporal two-alternative forced-choice (2AFC). The target was presented in one of the two-time intervals, whereas the flankers were always presented in both time intervals. Observers had to report in which time interval the target was presented. Feedback was provided for incorrect trials. The contrast threshold of the target was estimated according to 1 up/3 down staircase. Each block was completed after 120 trials or 14 reversals. Contrast thresholds were estimated by algebraic mean of the last 6 reversals. As in previous studies on MD, we used visual aids to facilitate the participants’ visual fixation with their PRL (Nilsson and Nilsson, 1986; Kasten et al., 2010; Rosengarth et al., 2013; Astle et al., 2015; Maniglia et al., 2018). Three red disks and a central dot were added on the visual display. Participants were instructed to keep fixation on the central dot. The three disks were displayed inside the scotoma, at the border with the intact part of the visual field. They had a diameter of 1° of visual angle and were positioned in an arrow-like configuration pointing toward the PRL (see Figure 3). Under correct ocular fixation, participants were therefore not able to perceive the disks (see Figure 3). Participants were informed that the red disks served as feedback on fixation. Should any disk become visible, participants were required to readjust their PRL toward the central dot. In this manner, the disks served as visual aids to facilitate fixation. The exact positions of the disks varied for each participant and were initially derived from the border of the retinal lesion observed in the OCT images and later adjusted by trial and error until the three of them completely disappeared inside the scotoma, although remaining as close as possible to its border. To make sure that the participants could see all the three Gabors in the training configuration, the global orientation of the triplets could be horizontal or vertical, depending on the size and the shape of the retinal lesion. Since the scotoma in the two eyes was very rarely overlapping in shape and size the training was conducted monocularly and only for the best eye. Before training began, participants were shown a prototypical training setup for each of the 4 conditions (3, 4, 6, and 8λ) with no time limit. Because the scotoma is often larger than the lesion visible on OCT, through participant feedback the placement of the target triplet and control discs was further adjusted.

Before each experiment, we also ran a practice block of 15 trials. The final training configuration remained unchanged throughout the experimental sessions.

#### 2.4.6 Collinear modulation

To measure the effects of training on collinear facilitation and inhibition, in the pre-and post-test measurement we estimated performances for both collinear and orthogonal (flankers rotated 90°) conditions. From these measurements, we then calculated the thresholds (TE) elevation as the log-ratio between the collinear and the orthogonal condition (Polat and Sagi, 1993; Maniglia et al., 2015), in which positive values indicate collinear inhibition and negative values indicate collinear facilitation. The testing was conducted monocularly and only for the trained eye.

### 2.5 tRNS stimulation

Participants in the PL + tRNS group (Stimulation) were trained with concomitant electrical brain stimulation over the occipital cortex, while participants in the control group (PL – only) performed the training with no stimulation. Pre, Mid, and Post-test were performed with no stimulation for all participants. To deliver the high-frequency tRNS, we used a battery-driven stimulator (BrainSTIM device, E.M.S., Bologna, Italy). While tRNS operates as a bidirectional alternating current, in our setup, the electrodes varied in size. The larger electrode (27 cm^2^), designated as the reference electrode, with lower current density, was placed over CZ. The active electrode (16 cm^2^) was positioned over OZ, covering the occipital cortex. The stimulation consisted of a randomly alternating current of 1.5 mA peak-to-peak intensity without offset. As in our previous study (Contemori et al., 2019), frequencies were distributed across a range of 100–640 Hz and with a maximal current density of 0.094 mA/cm^2^. Stimulation duration covered the whole training session (~25 min, see the ‘*Procedure*’ section). tRNS was only applied during training and not during tests. Thus, data analyzed for the PL – only and PL + tRNS groups were collected under the same experimental conditions.

### 2.6 Statistical analyses

Although participants were randomly assigned to the two groups, differences in the average severity of visual impairment between the two groups were expected due to the small sample size. To account for individual and group baseline differences, we represented data from the transfer tasks as a percentage of change (PE) from the baseline. We then conducted a statistical analysis to assess whether there was a significant difference in improvement between the two groups. PE was calculated as the ratio of the difference between mid (or post) minus pre-test: $\left(mid\;\mathrm{or}\;\mathrm{post}-pre\right)/pre$ (Kaiser, 1989; Maxwell and Howard, 2016; Jennings and Cribbie, 2022). This normalization allowed us to compare performances at the mid-test and the post-test relative to each participant’s baseline.

For the training task, we calculated the threshold elevation (TE) as the log-ratio between the collinear and orthogonal conditions (Polat and Sagi, 1993; Maniglia et al., 2015). Data were pre-processed and analyzed using the R statistical computing environment (R Core Team, 2012). Due to heterogeneity in variance between groups, parametric analysis was not suitable. Additionally, the longitudinal experimental paradigm requires consideration of the non-independence of measurements within subjects. Therefore, we conducted a nonparametric analysis of variance for both PE and TE.

To achieve this, we first aligned and ranked the data using the ‘art()’ function from the ‘ARTool’ package (Wobbrock et al., 2011). Statistical significance was assessed using mixed effects ANOVA on the transformed data, employing the ‘lmer()’ function from the ‘lme4’ package (Bates et al., 2015; Kuznetsova et al., 2017). The mixed-effects model included session and group as main effects, along with their interaction, and treated the participant as a random effect. The use of an Aligned Rank Transform (ART) ensured appropriate Type I error rates and sufficient power for main effects and interactions. This approach has been demonstrated to be robust when dealing with clustered data, repeated measures, and even missing or unbalanced data (Durner, 2019). For Bonferroni-corrected post-hoc pairwise comparisons, we implemented an extension of the ART procedure known as ART-C to avoid inflating Type I error rates (Elkin et al., 2021). ART-C allows for specific comparisons between levels of factors, and ARTool generates aligned-and-ranked responses tailored to those comparisons.

## 3 Results

This study aimed at testing whether occipital tRNS improves perceptual learning effects in participants with macular degeneration. In the following section, we first present the effects of stimulation on the trained task (collinear lateral masking). Next, we examine whether learning generalized to other tasks.

### 3.1 Training

**Figure 4:**
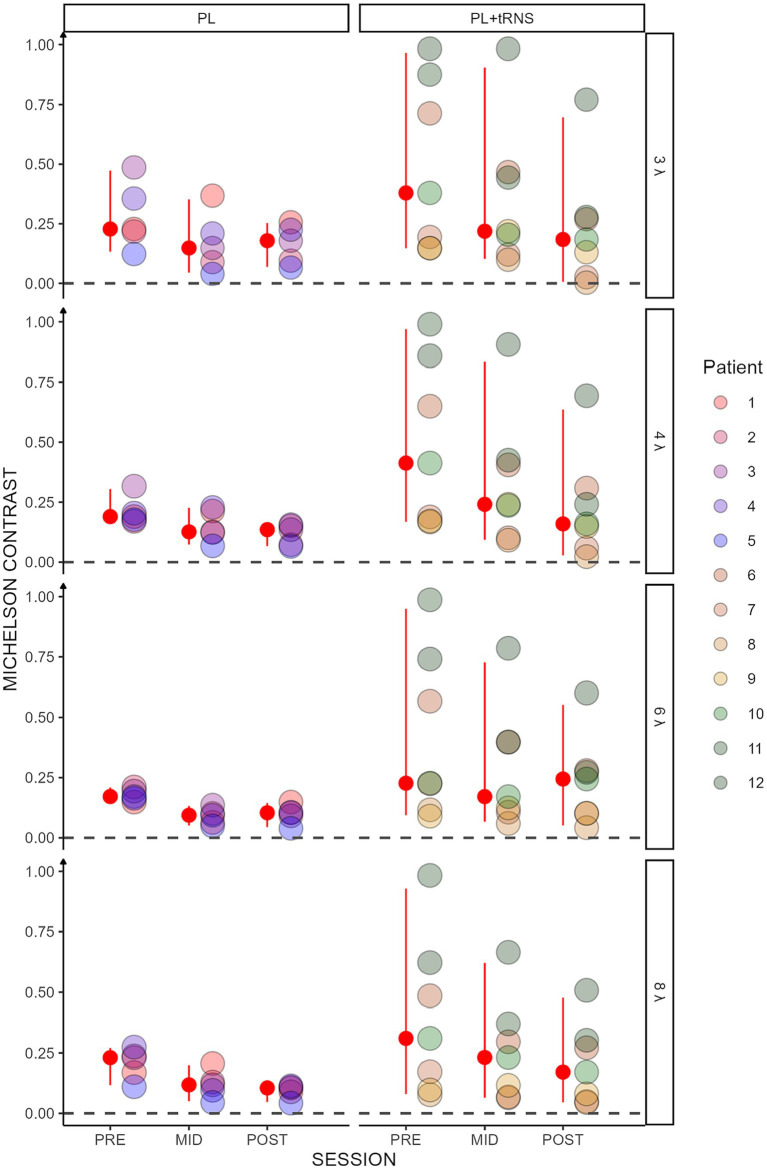
Contrast threshold in the trained task. Contrast thresholds plotted as a function of the session (pre, mid, post) for the two groups (‘PL – only’ on the left and ‘PL + tRNS’ on the right). Individual data points are presented for target-to-flanker separations of 3λ, 4λ, 6λ, and 8λ. The median is highlighted in red, along with the 2.5% lower and 97.5% upper quantiles. Lower Michelson contrast values correspond to heightened sensitivity.

Contrast thresholds for the collinear configuration are shown for the two groups (‘PL’ and ‘PL + tRNS’) in Figure 4. To test for significant effects of training on lateral masking, we conducted an Aligned Rank Transform Anova (ART Anova) with Kenward-Roger approximation on the contrast thresholds for the collinear condition, with main factors Group (PL + tRNS vs. PL – only), Target-to-flanker separation (3λ, 4λ, 6λ and 8λ), and Training (pre-, mid-, post-test). This ART ANOVA led to significant main effects of Training (*F*(2,110) = 76.800, *p* < 0.001, ηp^2^ = 0.58) and Target-to-flankers separation (*F*(3,110) = 7.511, *p* < 0.001, ηp^2^ = 0.17) but not of the Group (*F*(1,10) = 1.821, *p* = 0.207, ηp^2^ = 0.15). *Post hoc* comparisons showed that contrasts thresholds were reduced between pre-and mid-tests, mid-and post-tests and pre-and post-tests (MID – PRE = −22.025, se = 2.980, *t* = −7.391, *p* < 0.001; POST – MID = −14.661, se = 2.980, *t* = −4.920, *p* < 0.001; POST – PRE = −36.686, se = 2.980, *t* = −12.311, *p* < 0.001). Moreover, contrast thresholds were significantly higher for Target-to-flanker separation 3λ than 6λ and 8λ (3λ – 6λ = 11.543, se = 3.712, *t* = 3.109, *p* = 0.010; 3λ – 8λ = 14.624, se = 3.712, *t* = 3.939, *p* = 0.001), and significantly higher for 4λ than 6λ and 8λ (4λ – 6λ = 9.786, se = 3.712, *t* = 2.636, *p* = 0.029; 4λ – 8λ = 12.867, se = 3.712, *t* = 3.466, *p* = 0.004). Additionally, the Group x Training interaction was significant (*F*(2,110) = 10.450, *p* < 0.001, ηp^2^ = 0.16). *Post hoc* comparisons revealed significant reductions in contrast thresholds for the PL + tRNS group between pre-and mid-test, between mid-and post-test and between pre-and post-test (PL + tRNS, MID – PL + tRNS, PRE = −14.071, se = 4.714, *t* = −2.985, *p* < 0.05; PL + tRNS, POST – PL + tRNS, MID = −15.786, se = 4.714, *t* = −3.349, *p* < 0.05; PL + tRNS, POST – PL + tRNS, PRE = −29.857, se = 4.714, *t* = −6.334, *p* < 0.001). For the PL – only group, the difference between pre and mid-test and between pre-and post-test were also significant (PL – only, MID – PL – only, PRE = −36.450, se = 5.578, *t* = −6.535, *p* < 0.001; PL – only, POST – PL – only, PRE = −41.250, se = 5.578, *t* = −7.395, *p* < 0.001), but not the difference between mid-and post-test (*p* = 1.0). Results for the orthogonal condition showed no significant effect for Group (*F*(1,10) = 0.911, *p* = 0.362, ηp^2^ = 0.08) or Target-to-flankers separation (*F*(3,110) = 0.348, *p* = 0.791, ηp^2^ < 0.01). However, there was a significant effect of Training (*F*(2,110) = 73.347, *p* < 0.001, ηp^2^ = 0.57) but the Group x Training (*F*(2,110) = 0.617, *p* = 0.542, ηp^2^ = 0.01) or the Group x Target-to-flankers separation interactions (*F*(6,110) = 0.394, *p* = 0.882, ηp^2^ = 0.01) were not significant.

Overall, these analyses suggest that for collinear flankers, the training was more effective in the PL + tRNS group. This effect was not observed for orthogonal flankers.

**Figure 5:**
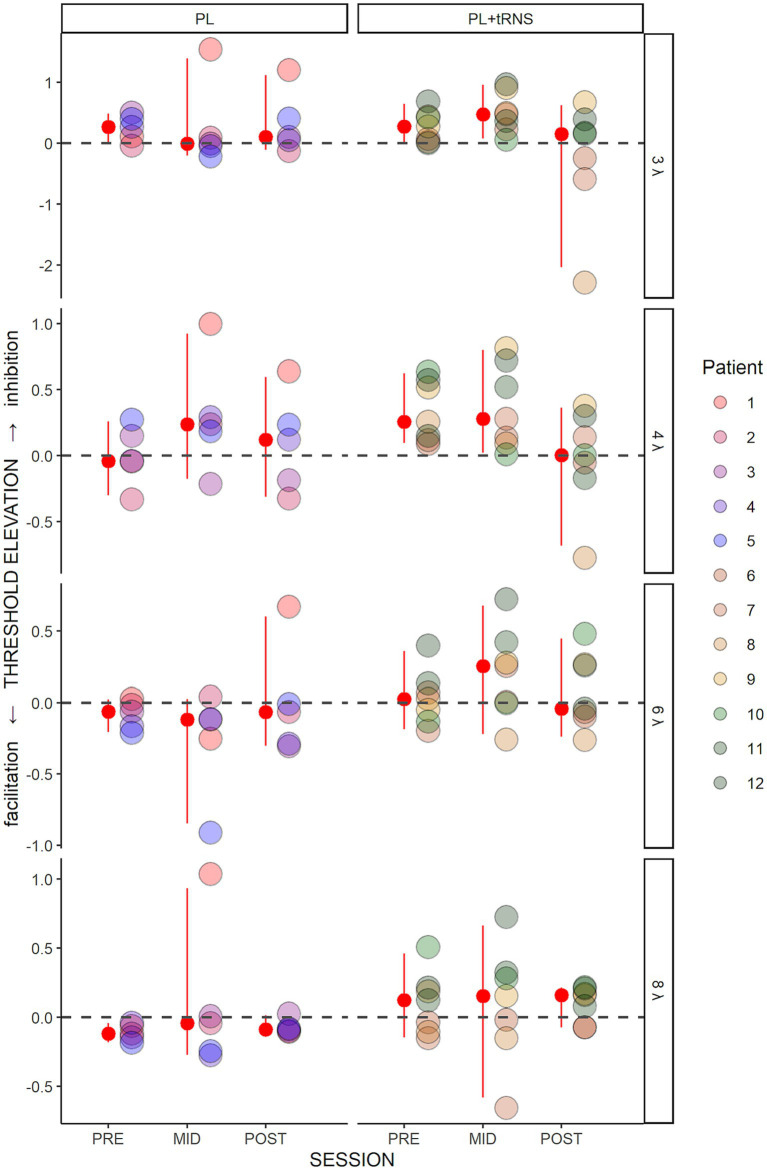
Threshold elevation (TE) in the trained task. Estimated variation in threshold elevation plotted as a function of the session (pre, mid, post) for the two groups (‘PL – only’ on the left and ‘PL + tRNS’ on the right). Individual data points are presented for target-to-flanker separations of 3λ, 4λ, 6λ, and 8λ. The median is highlighted in red, along with the 2.5% lower and 97.5% upper quantiles. Positive values signify inhibition, whereas negative values indicate facilitation.

Figure 5 shows the threshold elevations (TE) along the training for the two groups. A positive value means collinear inhibition while a negative value means collinear facilitation. An Aligned Rank Transform Anova (ART Anova) with Kenward-Roger approximation conducted on these data and including as factors the Group (PL – only vs. PL + tRNS) and Target-to-flankers separation (3λ, 4λ, 6λ, and 8λ), indicated that the effect of Group (*F*(1,10) = 1.117, *p* = 0.315, ηp^2^ = 0.10) was not significant. However, we found a significant effect of Session (*F*(2,110) = 3.323, *p* = 0.040, ηp^2^ = 0.06) and Target-to-flankers separation (*F*(3,110) = 4.477, *p* = 0.005, ηp^2^ = 0.11). Post-hoc comparisons revealed that contrast thresholds were higher in mid-test than in post-tests (POST – MID = −18.500, se = 7.432, *t* = −2.489, *p* = 0.043), but were not different between pre-and mid-tests or pre and post-tests (*p* > 0.05). The Anova also showed a significant Group x Session interaction (*F*(2,110) = 3.747, *p* = 0.027, ηp^2^ = 0.06). Pairwise comparisons for the PL + tRNS group revealed that TE at post-test was significantly lower than at mid-test (PL + tRNS, POST – PL + tRNS, MID = −32.286, se = 9.341, *t* = −3.456, *p* = 0.012), but no difference was found between pre-test and mid-test or between pre-and post-test (respectively *p* = 1.0 and *p* = 0.304). For the PL – only group there was no significant difference between pre-, mid-and post-test (*p* = 1.0).

These results suggest that tRNS over the occipital cortex induced a reduction of inhibition for low Target-to-flankers separations (3λ and 4λ). This effect was not observed in MD participants from the PL – only group. It should be noted that this result is consistent with a recent report regarding the effects of tDCS stimulation on MD patients using a similar training (Raveendran et al., 2021). In the next sections, we describe the effects of the training on other tasks (contrast sensitivity, near and far visual acuity, and visual crowding) to determine whether the learning transfers to untrained visual functions.

### 3.2 Contrast sensitivity

**Figure 6:**
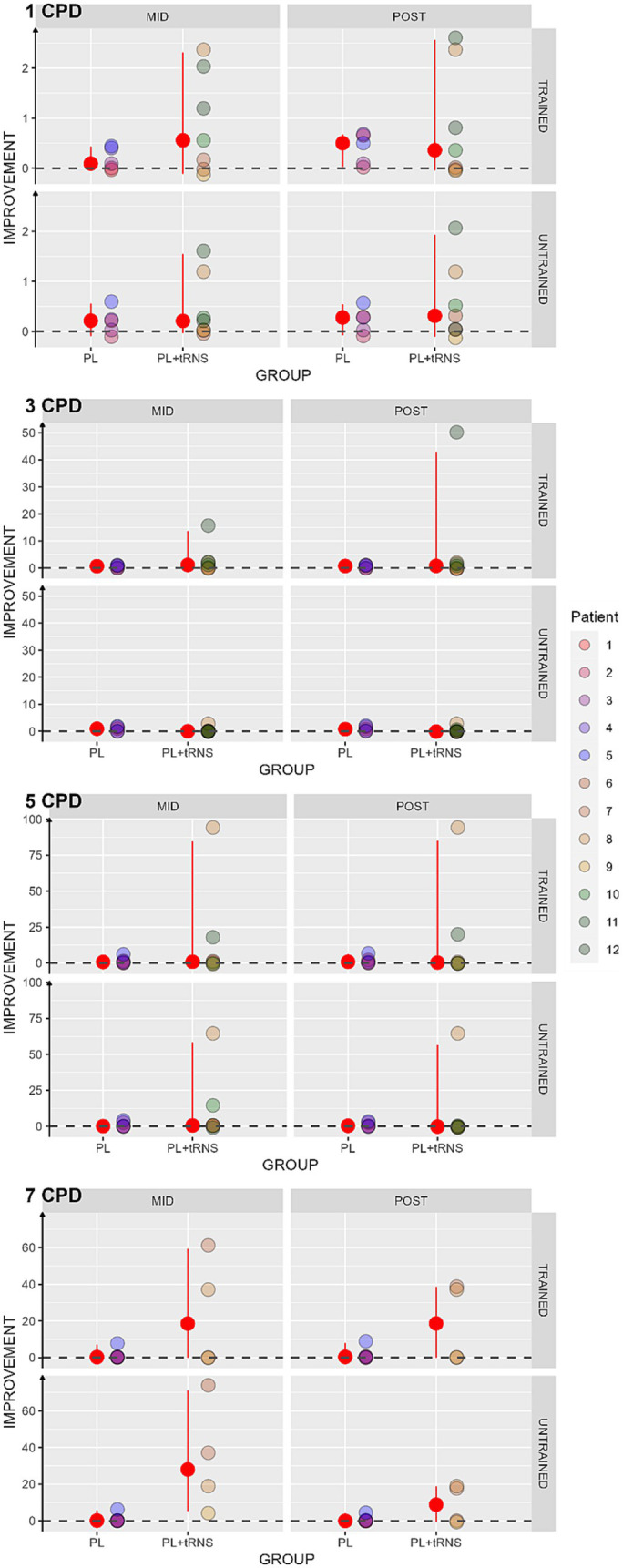
Percentage change scores in contrast sensitivity. Estimated percentage changes in contrast sensitivity, calculated as (mid or post – pre)/pre, and is presented as a function of the session (mid or post) for two distinct groups: ‘PL – only’ on the left and ‘PL + tRNS’ on the right. Individual data points obtained from both the trained and untrained eye are depicted for spatial frequencies of 1, 3, 5, and 7 cycles per degree (cpd). The median values are highlighted in red, accompanied by the 2.5% lower and 97.5% upper quantiles. The higher the value the better the improvement.

Contrast sensitivity was computed as log(1/contrast threshold). Then we calculated the percentage of score change for each subject as an improvement index (Kaiser, 1989; Berry and Ayers, 2006): $\left(mid\;\mathrm{or}\;\mathrm{post}-pre\right)/pre$ where pre, mid and post are the contrast sensitivity values at the pre-test, mid-test, and post-test, respectively. In this case, the higher the index, the better the improvement. An index of zero indicates no improvement. The results obtained in our two populations are shown in Figure 6. As several patients were not able to perform the task at mid-and post-test for the spatial frequency of 5 and 7 cpd, we considered in these cases that there was no improvement and replaced the missing values by zero. To avoid biasing the results, the Aligned Rank Transform Anova (ART Anova) with Kenward-Roger approximation was conducted separately for each Spatial frequency on the percentage change scores, with factors Group (PL + tRNS vs. PL – only), Eye (trained vs. untrained) and Session (mid vs. post). For the Spatial Frequency of 1 cpd, the Anova only showed a significant effect of Eye (*F*(1,30) = 6.345, *p* = 0.017, ηp^2^ = 0.17). Post-hoc analysis indicated a higher improvement for the trained eye than the untrained eye (Trained – Untrained = 4.386, se = 1.74, *t* = 2.519, *p* = 0.017). The ART Anova for the Spatial Frequency of 3 cpd showed a significant effect of the Session (*F*(1,30) = 13.629, *p* < 0.001, ηp^2^ = 0.31), Eye (*F*(1,30) = 15.586, *p* < 0.001, ηp^2^ = 0.34), the Session x Group interaction (*F*(1,30) = 10.597, *p* = 0.003, ηp^2^ = 0.26), the Session x Eye interaction (*F* (1,30) = 12.231, *p* = 0.001, ηp^2^ = 0.29) and the Group x Eye interaction (*F* (1,30) = 10.112, *p* = 0.003, ηp^2^ = 0.25). Post-hoc tests indicated a higher improvement at post-than mid-test (MID – POST = −9.371, se = 2.54, *t* = −3.692, *p* < 0.001), and a higher improvement for the trained eye (Trained – Untrained = 9.757, se = 2.47, *t* = 3.948, *p* < 0.001). However, post-hoc analyses for the different interactions showed no difference between mid-and post-test for neither of the two groups or neither of the two eyes. Moreover, the Anova carried out for the Spatial frequency of 5 cpd and 7 cpd showed no significant effect for none of the factors.

We found an increased contrast sensitivity for the spatial frequency of 1 and 3 cpd only, regardless of the group. We did not observe such an increase for the other spatial frequencies.

### 3.3 Far visual acuity

**Figure 7:**
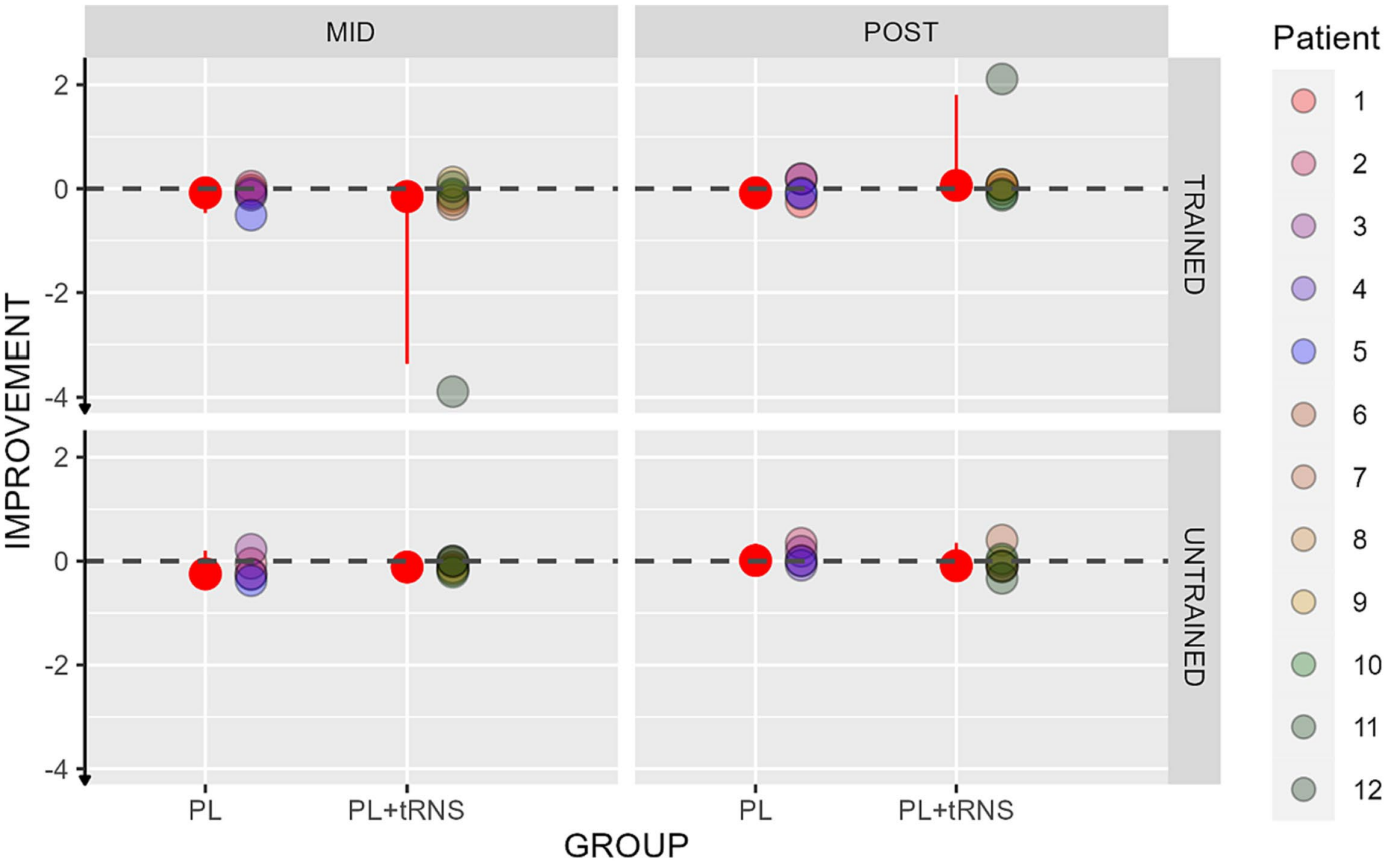
Percentage change scores for the Sloan visual acuity test. Percentage change scores for the Sloan visual acuity plotted as a function of the session (mid or post) for the two groups (‘PL – only’ on the left and ‘PL + tRNS’ on the right) in the trained and untrained eye. The median values are highlighted in red, accompanied by the 2.5% lower and 97.5% upper quantiles. The lower the value the better the improvement.

For visual acuity, the percentage change score was calculated upon the LogMar output from the FrACT Sloan test and can be interpreted as follows: a negative percentage change score reflects an improvement, an index of zero no change, and a positive index a worsening in the performance after the training. The values obtained in our two groups are shown in Figure 7. An Aligned Rank Transform Anova (ART Anova) with Kenward-Roger approximation conducted on ratio between the letter size at the mid-and post-test divided by the baseline at the pre-test data, including as factors Group (PL – only vs. PL + tRNS) and Eye (trained vs. untrained) and Session (mid vs. post) indicated that the effect of Group (*F*(1,10) = 0.709, *p* = 0.420, ηp^2^ = 0.07), Eye (*F*(1,30) = 0.153, *p* = 0.698, ηp^2^ < 0.01) and the Group x Eye interaction (*F*(1,30) = 0.263, *p* = 0.612, ηp^2^ < 0.01) were not significant. However, the effect of Session was significant (*F*(1,30) = 12.327, *p* = 0.001, ηp^2^ = 0.29). *Post hoc* comparisons for the group factor with the null hypothesis of zero mean showed that the average in both groups was not significantly different from zero (mean PL – only = −0.049, se = 0.147, *t* = −0.338, *p* = 0.742; mean PL + tRNS = −1.118, se = 0.124, *t* = −0.946, *p* = 0.732).

Overall, our analyses suggest that the training did not affect far visual acuity.

### 3.4 Near visual acuity

**Figure 8:**
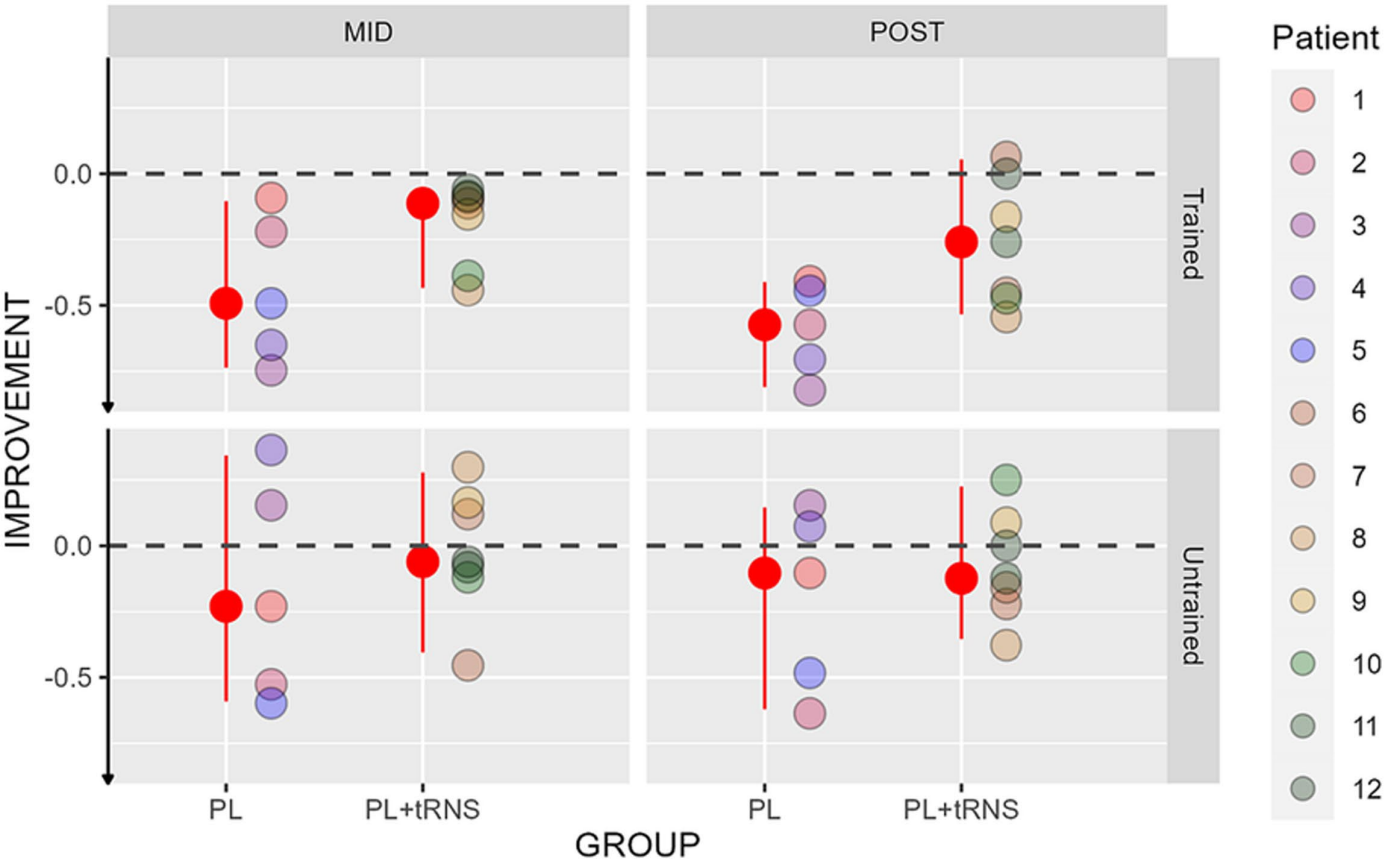
Percentage change scores for the near visual acuity test. Percentage change scores for the near visual acuity plotted as a function of the session (mid or post) for the two groups (‘PL – only’ on the left and ‘PL + tRNS’ on the right) in the trained and untrained eye. The median values are highlighted in red, accompanied by the 2.5% lower and 97.5% upper quantiles. The lower the value the better the improvement.

Improvements in near visual acuity are shown in Figure 8 for the two groups. An Aligned Rank Transform Anova (ART Anova) with Kenward-Roger approximation conducted on ratio between the letter size at the mid-and post-test divided by the baseline at the pre-test data, including as factors the group (PL – only vs. PL + tRNS) and the Eye (trained vs. untrained) and the Session (mid vs. post) indicated that both the effect of Group (*F*(1,10) = 5.872, *p* = 0.036, ηp^2^ = 0.37) and the effect of Eye (*F*(1,30) = 9.923, *p* = 0.004, ηp^2^ = 0.25) were significant, while the effect of Session (*F*(1,30) = 0.838, *p* = 0.367, ηp^2^ = 0.03), and the Group x Eye interaction (*F*(1,30) = 0.748, *p* = 0.394, ηp^2^ = 0.02) were not. *Post hoc* comparisons for the Group factor with the null hypothesis of zero mean showed that both groups differed from zero (mean PL – only = −0.349, se = 0.058, *t* = −5.985, *p* < 0.001; mean PL + tRNS = −0.137, se = 0.049, *t* = −2.777, *p* = 0.020). *Post hoc* comparisons for the eye factor with the null hypothesis of zero mean showed that both the trained and untrained eye differed from zero (mean trained = −0.370, se = 0.054, *t* = −6.857, *p* < 0.001; mean untrained = −0.116, se = 0.054, *t* = −2.142, *p* = 0.041).

Overall, our data suggest that training caused a reduction in performance (negative ratio) in both eyes.

### 3.5 Visual crowding

**Figure 9:**
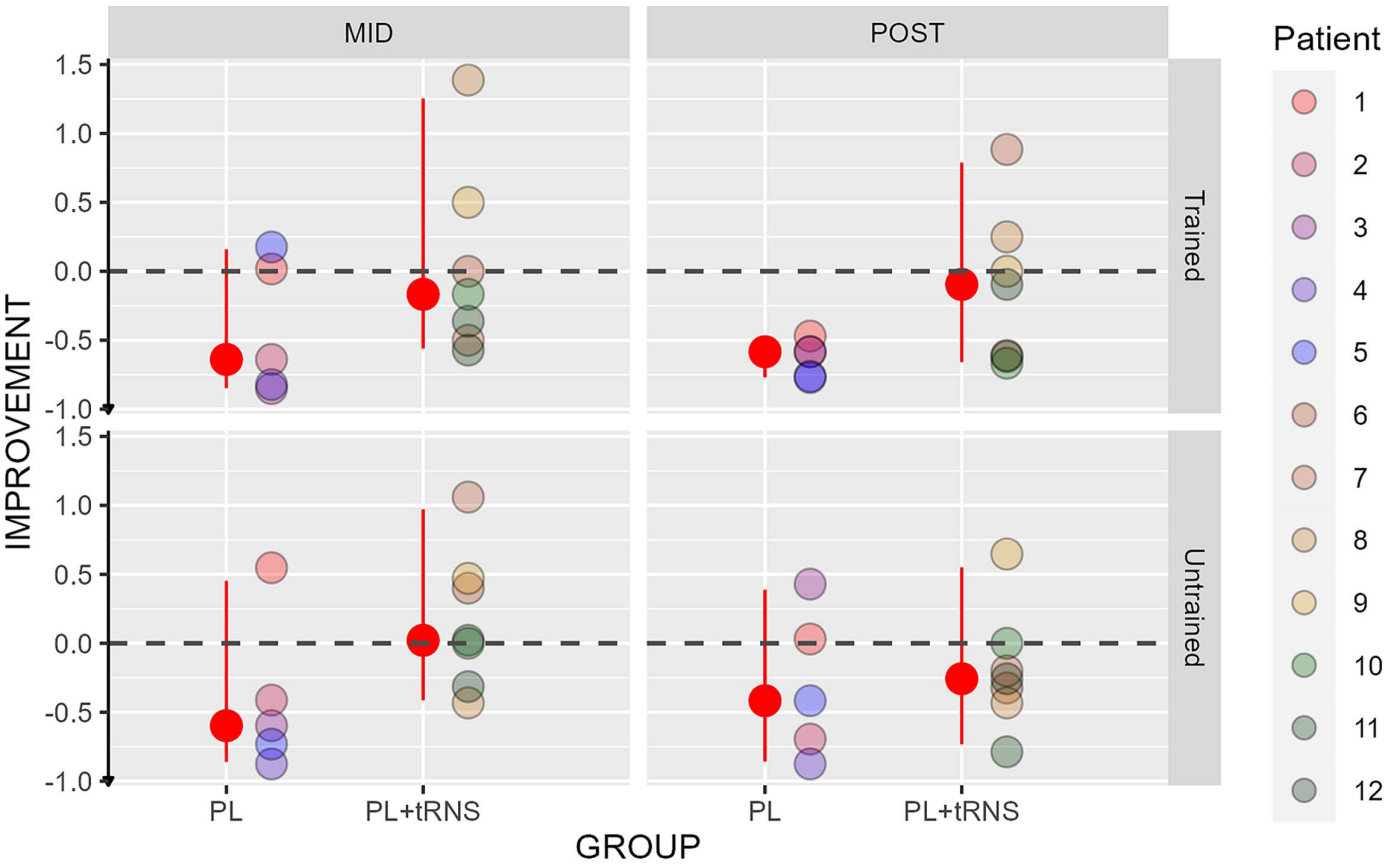
Percentage change scores for the crowding test. Percentage change scores for the Crowding plotted as a function of the session (mid or post) for the two groups (‘PL – only’ on the left and ‘PL + tRNS’ on the right) in the trained untrained eye. The median values are highlighted in red, accompanied by the 2.5% lower and 97.5% upper quantiles. The lower the value the better the improvement.

Improvements in the crowding tests are shown in Figure 9 for the two groups. An Aligned Rank Transform Anova (ART Anova) with Kenward-Roger approximation conducted on the percentage change scores, including as factors the group (PL – only vs. PL + tRNS) and the eye (trained vs. untrained) and the session (mid vs. post) indicated that the effect of Group was significant (*F*(1,10) = 5.394, *p* = 0.043, ηp^2^ = 0.3), while both the effects of Eye (*F*(1,30) = 0.390, *p* = 0.537, ηp^2^ = 0.01), and Session (*F*(1,30) = 0.738, *p* = 0.397, ηp^2^ = 0.02) were not. *Post hoc* comparisons for the Group factor with the null hypothesis of zero mean showed that only the improvement in the PL – only group, statistically differed from zero (mean PL – only = −1.444, se = 0.144, *t* = −0.79, *p* = 0.023; mean PL + tRNS = −0.0257, se = 0.122, *t* = 0.214, *p* = 0.838).

Overall, our data suggest that training caused a reduction of visual crowding but only in the PL – only group.

### 3.6 Transfer tasks

In the following table (Table 2) we summarized the results of the transfer tasks. Statistically significant results are highlighted in green.

**Table 2:** Summary of the results observed for the transfer tasks.

Group	Eye	CS	SF 1	SF 3	SF 5	SF 7	FVA	NVA	VC
PL – only	Trained	X	V	V	X	X	X	V	V
	Untrained	X	X	V	X	X	X	V	V
PL + tRNS	Trained	V	V	V	X	X	X	V	X
	Untrained	V	X	V	X	X	X	V	X

Green “Vs” symbolize significant results, while red “Xs” indicate non-significant ones. From left to right: contrast sensitivity (CS), spatial frequencies (SF 1, SF 3, SF 5, SF 7), far visual acuity with Sloan letters (FVA), near visual acuity (NVA), visual crowding (VC).

## 4 Discussion

In this study, we tested for the first time a training paradigm that combines perceptual learning and transcranial random noise stimulation (tRNS) in patients with MD. MD is the main cause of visual impairment in the western world, and with the aging population worldwide, it is bound to affect an increasing number of individuals every year. Perceptual learning (PL), the practice-induced improvement in perceptual abilities, has been successfully used in clinical settings (for a review, see Campana and Maniglia, 2015); however, its efficacy in MD participants is not clear. A possible reason might be the reduced neural plasticity in elderly populations. Recent studies showed that combining tRNS with PL has the potential to improve learning effects. tRNS is known to produce two main effects within the stimulated cortical area: a general increase in cortical excitability (Moret et al., 2018) and a resonance phenomenon between the externally induced noise and the stimulus-related signal (Miniussi et al., 2013; van der Groen and Wenderoth, 2016; Fertonani and Miniussi, 2017). Remedios et al. (2019) recently showed that at the cellular level, tRNS can modulate the activity of Na + channels. This modulation acts differently depending on the intensity of the current and the type of stimulated neurons, but at the optimal level, it could facilitate the processing of subthreshold stimuli (van der Groen and Wenderoth, 2016; Remedios et al., 2019). The repetitive activation of sodium channels and the associated influx of Na + ions inside the membrane (Fertonani et al., 2011; Ho et al., 2013) may interfere with the depressed state of the neurons, repolarizing their membranes near to its resting state and thereby preventing sensory adaptation due to the repeated visual stimulation (Campana and Maniglia, 2015). Sensory adaptation during visual training is known to be a limitation for the generalization of learning (Harris et al., 2012; Harris and Sagi, 2015). Reducing adaptation is thus a major issue for effective clinical applications.

Here, we trained 5 MD patients on a contrast detection task with lateral masking while they received concomitant occipital tRNS (the PL + tRNS group). A control group of 7 patients performed the training without stimulation (the PL – only group). Based on our previous studies, we expected an improvement in the trained task for both groups (Maniglia et al., 2018). We also hypothesized that stimulation would significantly boost learning effect in the ‘PL + tRNS group (Contemori et al., 2019). Moreover, we expected that tRNS improved generalization of learning to untrained visual tasks, as observed in studies using this technique in other clinical populations (Camilleri et al., 2014; Campana et al., 2014).

### 4.1 Trained task

Results showed a significant decrease in contrast thresholds after 12 training sessions for both groups. Between the 12th and the 24th training session, only the Stimulation group continued to improve while the PL – only group reached a plateau (see Figure 4). Besides, we did not observe any significant difference between the two groups when the flankers were orthogonal to the target, which could indicate a specific effect of stimulation on the collinear configuration. This hypothesis is supported by recent studies that found a specific reweighting of the perceptual field following occipital stimulation (Battaglini et al., 2019; Raveendran et al., 2020).

In terms of threshold elevations (TE), which is a measure of the ratio between collinear facilitation and inhibition, we found a significant effect of target-to-flanker separation and a significant interaction between groups and sessions. Post-hoc analyses revealed that only the Stimulation group had a significant modulation of the lateral interaction after the training (i.e., at the post-test). As can be observed in Figure 5, the TE as a function of target-to-flankers separation, in the pre-and mid-test, is first positive (inhibition) and becomes negative (facilitation) after training. This result is in agreement with previous studies that found an increase in facilitation and a reduction of inhibition after training (Maniglia et al., 2016, 2018). In our case, however, the modulation of lateral interactions reaches significance only for the Stimulation group, indicating that stimulation played a fundamental part in this process. Previous studies suggested that the effect of occipital stimulation is to temporarily weaken the strength of lateral connections (Battaglini et al., 2019; Raveendran et al., 2020). Our data suggest that this initial weakening may in turn facilitate the long-term reshaping of the horizontal connections promoted by training.

It should be noted that there was a significant interindividual variability in the Stimulation group, which could be explained in different ways. The sensitivity to the stimulation, for example, may differ from one patient to another. Additionally, it is possible that the placement of the electrodes was not optimal for all participants because important variabilities exist in the positions and extents of visual areas within the occipital cortex (Dougherty et al., 2023). Moreover, an important limitation of our study is the absence of a sham group. Given our long experimental protocol (training lasted several weeks), it was difficult to recruit a larger cohort and spreading our 12 patients into three groups to add a sham group would have severely reduced the statistical reliability of our analyses. To address this issue in future studies, it will be important to find ways to increase the number of patients, for example with the implementation of a multicentric protocol. Furthermore, it should be noted that all patients exhibit different clinical characteristics. To address whether performance during training related to individual differences such as the size of the scotoma or visual acuity, we computed Pearson correlation coefficients (see Supplementary materials). No significant correlation was found.

### 4.2 Transfer tasks

We confirmed results from previous studies using perceptual learning in MD (Maniglia et al., 2016, 2018) which found that training transferred to near visual acuity for both the trained and the untrained eye. Unexpectedly, we did not find transfer to far visual acuity, and transfer to visual crowding was limited to the PL – only group. Similarly, no significant transfer to CS at high spatial frequencies (5 and 7 cpd) was found in neither of the two eyes. Moreover, we did not find significant differences between the mid-tests and the post-tests for the two groups. It suggests that 12 sessions could be enough to trigger the transfer. In the following paragraphs, we will discuss these results in more detail.

#### 4.2.1 Contrast sensitivity

Among the different transfer tasks, contrast sensitivity is certainly the closest to the trained task. The fundamental difference is the absence of the two flankers and therefore the absence of contextual influences. It was previously shown that the improvement in contrast sensitivity achieved by training with lateral masking could also transfer to untrained spatial frequencies (Sagi, 2011). This transfer is more likely to pass from lower frequencies to higher ones rather than the opposite (see Sagi, 2011 for a review). Visual processing in MD patients is much less affected for low spatial frequencies than for higher ones (Musel et al., 2011; Sagi, 2011; Peyrin et al., 2017; Ramanoël et al., 2018). Training directly with medium and/or high spatial frequencies is not always possible since the task could be too difficult, and the patient could be frustrated by failure. A transfer to higher spatial frequencies is therefore a desirable property of perceptual learning with lateral masking. The results of the contrast detection test show that the Stimulation group improved more than the PL – only group but only for low spatial frequencies (3 cpd). This finding is inconsistent with previous results which demonstrated that tRNS enhances the detection of an isolated Gabor only when its spatial frequency is high (12 cpd) (Battaglini et al., 2019). The reason could be related to the fact that some participants were not able to perform the task at mid-and post-test for the spatial frequency of 5 cpd and 7 cpd. In these cases, we considered that there was no improvement. Future studies with a larger number of patients will be needed to clarify this point.

#### 4.2.2 Near and far visual acuity

Previous studies reported improvement in visual acuity following training with lateral masking (Polat, 2009; Camilleri et al., 2014). In our data, training transferred to NVA but did not produce an improvement in FVA with Sloan letters. This seemingly contrasting result can have multiple explanations. First, the background and stimuli luminance in the two tasks were reversed and this could have had an impact on the tests. Patients with MD often suffer from visual glare (Hogg and Chakravarthy, 2006). In the FVA test with the FrACT software, the letters presented were black on a white background, while in the NVA test on Matlab, the letters were white on a black background. Some of the participants reported difficulties in the first of the two tests due to the bright background color. The glare effect reported by the subjects was not constant during the measurements but was rather growing over time. This may have affected the measurement by reducing its reliability. A second reason could be related to the different duration of the stimuli. During the FrACT test, some participants reported seeing the letter in the first few moments after the onset of the stimuli, but the attempt to keep a prolonged fixation produced a later distortion of their perception. This might be due to some habituation effects compensated by large ocular drifts (Déruciz et al., 2004). The short stimulus duration in the NVA test may have prevented this problem. The last possibility is that given the temporal structure of the task, the training was especially effective over some transient visual channels and less over sustained ones, thereby producing a greater benefit to the tachistoscopic NVA test (Tolhurst, 1975). This last possibility opens an interesting direction for future studies aiming at optimizing training effects.

#### 4.2.3 Visual crowding

In the visual crowding task, we found a reduction in the critical space for the PL – only group, but not for the Stimulation group. This result is surprising if we consider that in the trained task, as well as in the contrast sensitivity task, there is an advantage of the Stimulation over the PL – only group. Also, it is in apparent contrast with the results of previous studies in which tRNS further reduced the critical distance during a perceptual learning regimen (Contemori et al., 2019). It is important to notice that in Contemori et al. (2019), the gain produced by tRNS was only observed when crowding was trained online during stimulation and not when it was tested as a transfer task. Moreover, another very recent study that tested the effect of repeated tRNS in adult amblyopes also found transfer of learning to visual acuity and contrast sensitivity, but only a limited transfer to crowded visual acuity (Donkor et al., 2021). The reason why tRNS has only limited transfer to crowding is still unclear and deserves further investigation.

### 4.3 Study limitations

The main limitation of our study is related to sample size and variability. Originally, we intended to include an independent sham stimulation group, with participants randomly assigned to either the experimental or sham group. However, despite considering numerous patients for study participation, only a limited number met the inclusion criteria for the tRNS stimulation. We also carefully applied exclusion criteria to filter out unstable patients, those with important comorbidities, or those under medication that could interact with the PL effect. Despite our effort, it was extremely challenging to include a large number of participants in the final sample. Consequently, we opted to re-analyze data previously collected using the same methodology as a control.

Although the control group data had been previously described in a prior publication (Maniglia et al., 2018), both the experimental and control groups were collected sequentially with temporal continuity. The entire data collection process was conducted by the same research team and utilized the same experimental setup. However, due to the extended duration of data collection, we cannot entirely rule out the possibility of environmental factors influencing potential differences between the groups and impacting the observed training effect.

It should also be noted that the use of various thresholding procedures complicates task comparisons. Initially, our focus was not on this comparison but on comparing groups within the same task. To ensure reliability, we repurposed tests from our previous work, which had demonstrated robust effects (Maniglia et al., 2016; Barollo et al., 2017; Maniglia et al., 2018; Contemori et al., 2019). On the other hand, this approach resulted in heterogeneity in thresholding procedures. Possibly, Some transfer tests may be more sensitive than others and thus lead to greater training related changes.

Lastly, In this study, we employed a stimulation intensity of 1.5 mA peak-to-peak, following the methodology outlined in Fertonani et al. (2011) and Contemori et al. (2019). While this intensity was commonly used in previous works, a more recent study (Van der Groen et al., 2022), suggested potential benefits in adopting a personalized intensity for each participant.

## 5 Conclusion

The combination of perceptual learning and tRNS led to greater improvements in the trained task (contrast detection) than perceptual learning alone. tRNS also induced larger transfer to untrained spatial frequencies. Furthermore, both groups showed transfer of learning to near visual acuity, but only the PL – only group showed in visual crowding. In conclusion, our study suggests that tRNS can be effectively combined with perceptual learning to improve vision in patients with macular degeneration. Further studies are however needed to fine-tune the stimulation parameters so as to maximize the efficacy of this approach.

## Data availability statement

The raw data supporting the conclusions of this article will be made available by the authors, without undue reservation.

## Ethics statement

The studies involving humans were approved by CNRS ethical committee (Comité de Protection des Personnes, protocole 13018). The studies were conducted in accordance with the local legislation and institutional requirements. The participants provided their written informed consent to participate in this study.

## Author contributions

GC: Conceptualization, Funding acquisition, Data curation, Formal analysis, Investigation, Methodology, Software, Visualization, Writing – original draft, Writing – review & editing. MM: Conceptualization, Data curation, Formal analysis, Investigation, Methodology, Software, Visualization, Writing – original draft, Writing – review & editing. JG: Data curation, Formal analysis, Investigation, Writing – review & editing. VS: Conceptualization, Funding acquisition, Investigation, Methodology, Resources, Writing – review & editing. MC: Investigation, Data curation, Writing – review & editing. BRC: Conceptualization, Funding acquisition, Methodology, Project administration, Supervision, Validation, Writing – review & editing. YT: Conceptualization, Funding acquisition, Methodology, Project administration, Resources, Supervision, Validation, Writing – review & editing.
