# Supplementary material for: tRNS boosts visual perceptual learning in participants with bilateral macular degeneration
Source: Front Aging Neurosci. 2024 Feb 21;16:1326435. doi: 10.3389/fnagi.2024.1326435 (PMC10914974; doi:10.3389/fnagi.2024.1326435)
Supplement: Supplementary file 1 [file Data_Sheet_1.docx]

**tRNS boosts Visual Perceptual Learning in Participants with Bilateral Macular Degeneration**

**Supplementary materials**

**Correlations between training and individual differences in scotoma size and visual acuity**

Patients with macular degeneration are characterized by large heterogeneity in clinical characteristics. To address whether performance during training related to individual characteristics such as the size of the scotoma or visual acuity, we computed Pearson correlation coefficients.

**Scotoma size**

There is evidence that visual performance deteriorates with increasing scotoma size. As a first test, we correlated scotoma size with contrasts thresholds measured during pre-tests in the collinear condition, regardless of the target-to-flanker separation. We did not observe significant correlations (3λ : r(10) = 0.008, p = 0.980 ; 4λ: r(10) = -0.041, p = 0.900 ; 6λ: r(10) = -0.061, p = 0.852 ; 8λ: r(10) = -0.099, p = 0.759). Similarly, no significant correlations were found with the contrast thresholds measured during mid-tests (3λ : r(10) = -0.094, p = 0.772 ; 4λ: r(10) = -0.173, p = 0.590 ; 6λ: r(10) = -0.045, p = 0.889 ; 8λ: r(10) = -0.050, p = 0.877) and post-tests (3λ : r(10) = -0.144, p = 0.655 ; 4λ: r(10) = -0.127, p = 0.694 ; 6λ: r(10) = -0.058, p = 0.858 ; 8λ: r(10) = -0.097, p = 0.764), see figure S1.


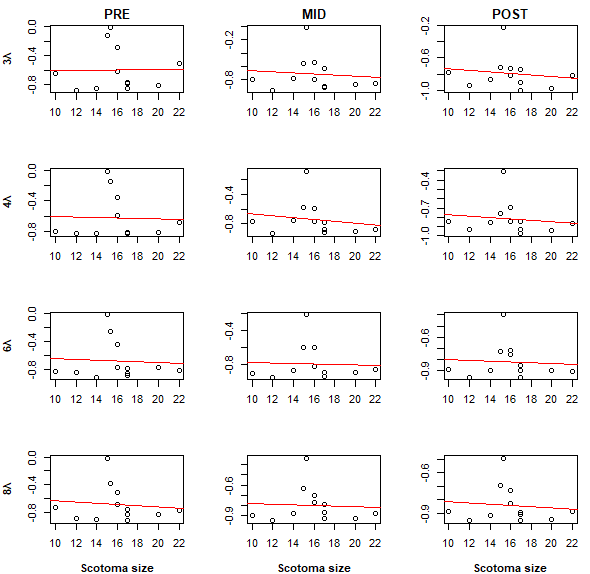


Figure S1. Correlations between the contrasts thresholds and the scotoma size (in degrees) in the collinear condition. No correlation is significant.

*.*

Results for the orthogonal condition were similar, with no significant correlation between the measured contrasts and the target-to-flankers separation during pre-tests (3λ : r(10) = -0.022, p = 0.947 ; 4λ: r(10) = -0.007, p = 0.982 ; 6λ: r(10) = -0.050, p = 0.878 ; 8λ: r(10) = -0.041, p = 0.900), mid-tests (3λ : r(10) = -0.190, p = 0.555 ; 4λ: r(10) = -0.031, p = 0.923 ; 6λ: r(10) = -0.073, p = 0.823 ; 8λ: r(10) = 0.011, p = 0.973), or post-tests (3λ : r(10) = -0.119, p = 0.714 ; 4λ: r(10) = -0.038, p = 0.908 ; 6λ: r(10) = -0.049, p = 0.880 ; 8λ: r(10) = -0.111, p = 0.732), see figure S2.


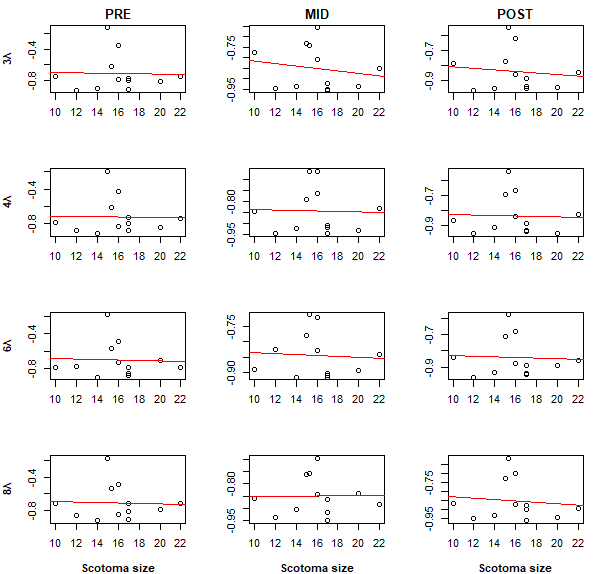


Figure S2. Correlations between the contrasts thresholds and the scotoma size (in degrees) in the orthogonal condition. No correlation is significant.

**Visual acuity**

No correlation was found between the visual acuity and the contrasts thresholds measured in the collinear condition during the pre-test (3λ : r(10) = 0.023, p = 0.944 ; 4λ: r(10) = -0.002, p = 0.995 ; 6λ: r(10) = 0.041, p = 0.900 ; 8λ: r(10) = 0.124, p = 0.700), mid-test (3λ : r(10) = -0.184, p = 0.567 ; 4λ: r(10) = -0.152, p = 0.638 ; 6λ: r(10) = -0.144, p = 0.655 ; 8λ: r(10) = -0.238, p = 0.456) or post-tests (3λ : r(10) = -0.172, p = 0.592; 4λ: r(10) = -0.180, p = 0.575 ; 6λ: r(10) = -0.242, p = 0.448 ; 8λ: r(10) = -0.167, p = 0.604), see figure S3.


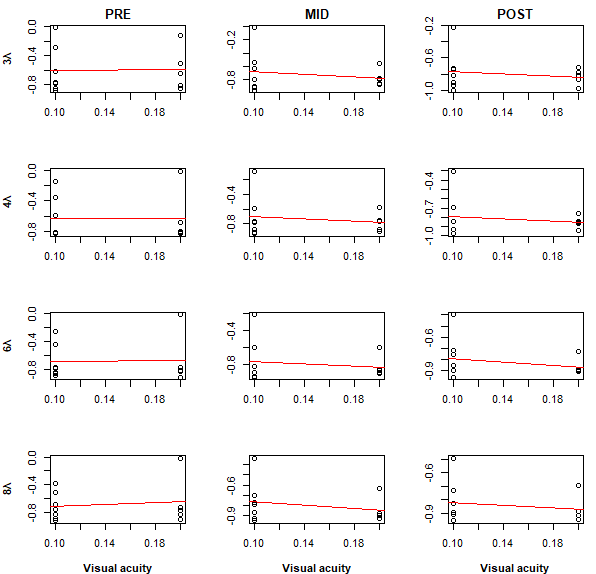


Figure S3. Correlations between the contrasts thresholds and the visual acuity in the collinear condition.

*No correlation was found between the contrasts thresholds measured for the four target-to-flanker separations used during the training (3λ, 4λ, 6λ and 8λ) during the pre-tests (left column), mid-tests (central column) or post-tests (right column).*

Similar results were obtained in the orthogonal condition (pre-tests: 3λ : r(10) = 0.153, p = 0.635; 4λ: r(10) = 0.100, p = 0.758 ; 6λ: r(10) = 0.138, p = 0.668 ; 8λ: r(10) = 0.183, p = 0.568; mid-tests: 3λ : r(10) = 0.039, p = 0.905; 4λ: r(10) = -0.171, p = 0.596 ; 6λ: r(10) = -0.230, p = 0.471 ; 8λ: r(10) = 0.009, p = 0.978; post-tests: 3λ : r(10) = -0.139, p = 0.667; 4λ: r(10) = -0.107, p = 0.740 ; 6λ: r(10) = -0.040, p = 0.903 ; 8λ: r(10) = -0.158, p = 0.624), see figure S4.


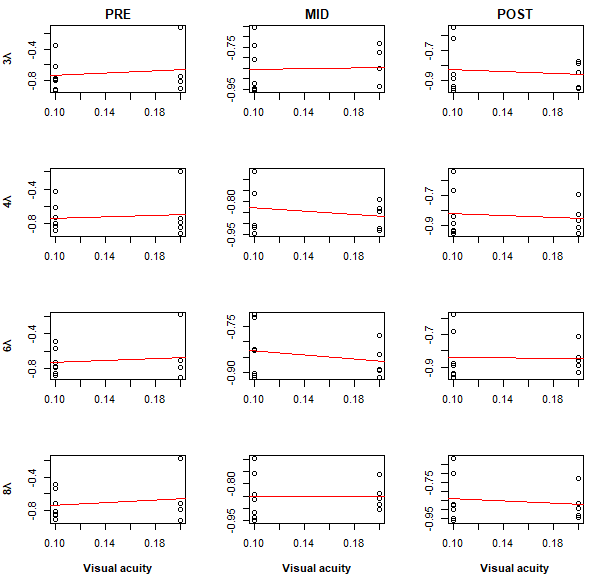


Figure S4. Correlations between the contrasts thresholds and the visual acuity in the orthogonal condition. No correlation is significant.
